# A possible new approach in the prediction of late gestational hypertension: The role of the fetal aortic intima-media thickness

**DOI:** 10.1097/MD.0000000000005515

**Published:** 2017-01-13

**Authors:** Silvia Visentin, Ambrogio P. Londero, Martina Camerin, Enrico Grisan, Erich Cosmi

**Affiliations:** a Department of Woman's and Child's Health, University of Padua, Padua; b Unit of Obstetrics and Gynecology, S. Polo Hospital, Monfalcone; c Department of Information Engineering, University of Padua, Padua, Italy.

**Keywords:** fetal abdominal aorta intima-media thickness, late-gestational hypertension, predictive markers

## Abstract

The aim was to determine the predictive role of combined screening for late-onset gestational hypertension by fetal ultrasound measurements, third trimester uterine arteries (UtAs) Doppler imaging, and maternal history. This prospective study on singleton pregnancies was conducted at the tertiary center of Maternal and Fetal Medicine of the University of Padua during the period between January 2012 and December 2014. Ultrasound examination (fetal biometry, fetal wellbeing, maternal Doppler study, fetal abdominal aorta intima-media thickness [aIMT], and fetal kidney volumes), clinical data (mother age, prepregnancy body mass index [BMI], and parity), and pregnancy outcomes were collected. The *P* value <0.05 was defined significant considering a 2-sided alternative hypothesis. The distribution normality of variables were assessed using Kolmogorov–Smirnoff test. Data were presented by mean (±standard deviation), median and interquartile range, or percentage and absolute values. We considered data from 1381 ultrasound examinations at 29 to 32 weeks’ gestation, and in 73 cases late gestational hypertension developed after 34 weeks’ gestation. The final multivariate model found that fetal aIMT as well as fetal umbilical artery pulsatility index (PI), maternal age, maternal prepregnacy BMI, parity, and mean PI of maternal UtAs, assessed at ultrasound examination of 29 to 32 weeks’ gestation, were significant and independent predictors for the development of gestational hypertension after 34 weeks’ gestation. The area under the curve of the model was 81.07% (95% confidence interval, 75.83%–86.32%). A nomogram was developed starting from multivariate logistic regression coefficients. Late-gestational hypertension could be independently predicted by fetal aIMT assessment at 29 to 32 weeks’ gestation, ultrasound Doppler waveforms, and maternal clinical parameters.

## Abstract 2

Supplemental Digital Content is available in the text

## 1 Introduction

The hypertensive disorders of pregnancy (HDP) account for a significant number of maternal and fetal deaths worldwide, affecting about 5% to 7% of pregnancies considered to be low risk and of which incidence reaches up to 20% in high-risk pregnancies. Late onset after 34 weeks’ gestation represents 5% to 17% of these.^[1]^ Despite the low prevalence of HDP, they are associated with a significant fetal neonatal and maternal mortality and morbidity, including an increased risk of maternal hypertension in later life.^[2,3]^ The syndrome etiology remains unclear. The most widely accepted theory postulates that a defective process of trophoblastic invasion in the uterine spiral arteries creates a localized hypoxic environment, having as physiological response the release of several factors that are hazardous to the systemic vascular endothelium, including reactive oxygen species, which have a harmful effect on the mother's systemic arterial endothelium.^[4]^

In recently published studies, an increased prevalence of signs of vascular damage in fetuses from pregnancies affected by preeclampsia or late-onset HDP emerged; aorta intima-media thickness (aIMT) was significantly increased in fetuses whose mothers developed HDP, in particular at 29 to 32 weeks’ gestation.^[5,6]^ Gestational hypertension should be considered as one of the adverse early risk factors that might predispose to an impaired fetal endothelial development during intrauterine life and cardiovascular events during adulthood. Nowadays, aIMT is a feasible, accurate, and sensitive marker of vascular remodeling, both in fetuses and children. When thicker, aIMT might be the result of poor intrauterine environment, as described in presence of intrauterine growth restriction (IUGR).^[5]^ Although several risk factors for preeclampsia have been recognized, individually they perform poorly from a screening perspective with a low positive predictive value.^[7,8]^ There is evidence that late-onset HDP compared with early-onset disease is more likely to be considered a consequence of underlying maternal vascular disease or metabolic imbalance, resulting in the deterioration of placental function closer to term.^[9–11]^ An abnormal uterine artery (UtA) Doppler velocimetry persistent from second trimester up to at 29 to 32 weeks’ gestation is considered to be a surrogate marker of chronic uteroplacental ischemia and an arise of umbilical artery (UA) pulsatility index (PI) a sign of fetal compromise.^[12]^

The aim of this study was to investigate the prediction of late-onset gestational hypertension by fetal ultrasound measurements, including aIMT, mean UtA PI, and maternal data collected during scan examination at 29 to 32 weeks’ gestation.

## 2 Materials and methods

This was a prospective observational study for late gestational hypertension carried out between January 2012 and December 2014 among patients attending for antenatal care at the University Hospital of Padua (tertiary referral center). We included in the study all singleton pregnancies that delivered in the University Clinic and performed a third trimester hospital scan to assess fetal growth, maternal–fetal Doppler evaluation, and fetal aorta intima media thickness assessment. Delivery before 34 weeks’ gestation, prepregnancy hypertensive disorders, HDP disorders diagnosed before the 34th week gestation, IUGR condition (defined by fetal weight below the 10th percentile, fetal abdominal circumference below the 10th percentile, or third trimester flattening of the growth curve with UA Doppler alterations, as PI > 2 standard deviation),^[13]^ cholestasis, hemolysis, elevated liver enzymes, low platelet count syndrome, pregestational diabetes, gestational diabetes, multiple pregnancies, major fetal congenital anomalies, pregnancies complicated by maternal history of cardiovascular disease or endocrine disorders such as thyroid and adrenal problems, severe obesity, and clinical chorioamnionitis were considered as exclusion criteria. The ethical committee of the hospital approved the study (P1826, in 2009). Furthermore, the present study was conducted in accordance with Helsinki Declaration, and written consent was obtained from the patients.

In June 2015, all the considered patients had delivered. Information about personal, obstetrical, and familial history were collected. In addition, demographic data (age, ethnicity, and parity), lifestyle variables (diet, physical activity, blood pressure, smoking, use of alcohol, or drugs), and risk factors for several diseases (obesity, diabetes mellitus, thrombophilia, HDP, preterm delivery, and IUGR) were also entered into a computer database. Further information about hospitalization and delivery (e.g., development of late hypertension, gestational age at delivery, mode of delivery, and neonatal data) were gathered from hospital maternity records. Patients affected by HDP were treated using methyldopa or nifedipine.

Twenty patients took part in another study about cardiovascular and renal risk in fetuses of HDP.^[5]^ Gestational age was calculated starting from the last known menstrual period and confirmed by ultrasound dating before 20 weeks of gestation.

Body mass index (BMI) was calculated (kg/m^2^) after measuring maternal height and weight. Maternal blood pressure was measured as previously described using an automated device (Myndraj Biomedical Elettronics IMP/9800, Burnaby, British Columbia, Canada).^[5]^ The women were in the seated position, their arms were supported at the level of the heart, using an adult cuff depending on the mid-arm circumference. We calculated the mean arterial pressure as the average of the last 3 stable measurements.^[14]^

The definitions of gestational hypertension and preeclampsia were those of the International Society for the Study of Hypertension in Pregnancy. In gestational hypertension, the systolic blood pressure should be 140 mm Hg or more and the diastolic blood pressure should be 90 mm Hg or more on at least 2 occasions 4 hours apart, developing after 20 weeks of gestation in previously normotensive women in the absence of significant proteinuria. In preeclampsia, there should be gestational hypertension with proteinuria of 300 mg or more in 24 hours, or 2 readings of at least ++ on dipstick analysis of midstream or catheter urine specimens if no 24-hour collection is available.^[15]^

For the present study, we considered small for gestational age (SGA) the newborns with a birth weight less than the 10th percentile. All infants with a birth weight above the 90th percentile according to gestational age were considered large for gestational age (LGA).^[16]^

### 2.1 Ultrasonographic examination

Two skilled operators (SV and EC) performed all the ultrasound scans using an ultrasound machine equipped with a 3.5- to 5-MHz linear array transducer (Voluson E8, GE Medical Systems, Chicago, IL, USA).

During the examination at 29 to 32 weeks’ gestation, fetal growth, fetal wellbeing, and maternal Doppler evaluation were assessed. The following fetal abdominal aorta characteristics were considered: fetal aIMT, fetal abdominal aorta diameter, and abdominal aorta PI. In addition, other ultrasound measurements were considered: UA PI, middle cerebral artery PI, kidneys’ mean volume, mean maternal UtA PI, mean UtA resistance index (RI), and the presence of bilateral notching of the UtAs. Fetal renal volumes were calculated by means of the following ellipsoid approximation formula: volume = length × width × thickness × 0.5233.^[5,17]^ The average volume of the 2 kidneys was used, and the volume percentile was calculated according to previous published charts.^[18]^

Intima-media thickness and diameter were measured in a coronal or sagittal view of the fetus at the dorsal arterial wall of the most distal 15 mm of the abdominal aorta sampled below the renal arteries and above the iliac arteries as previously described; gain settings were used to optimize image quality. Abdominal aIMT was defined as the distance between the leading edge of the blood–intima interface and the leading edge of the media–adventitia interface on the far wall of the vessel. These measurements were taken, and the arithmetic mean aIMT aortic intima media thickness was considered for the study. Aortic diameter was measured at the same level of aIMT, from inner wall to the wall edges. All images were taken at end-diastole of the cardiac cycle to minimize the variability. End-diastole was determined using the cine-loop capability of the ultrasound machine once the images of the entire cardiac cycle were frozen. An angle as close as possible to 0 was achieved between the Doppler ultrasound beam and the direction of blood flow in each vessel. The high-pass filter was set at 100 Hz.^[19]^

The aIMT intraobserver and interobserver agreements were previously assessed and found to be both greater than 0.85.^[5,19]^ Furthermore, the aIMT images were saved and then analyzed also off-line by a specific software, that was previously found to significantly correlate with manual measurements with a coefficient of 0.9.^[20]^ All manual measurements of the present study were confirmed by this off-line automated wall tracking software.^[20]^ The UA was evaluated in a free loop of the umbilical cord. Each measurement was taken during fetal apnea after 3 consecutive, similar waveforms were obtained. PI and time-averaged velocities (defined as the area under the velocity spectral envelope) then were measured using the machine software.^[19]^ The Doppler velocimetry of UtAs was performed with a convex probe with 3.5 MHz of frequency. Color flow mapping was used to identify in turn left and right UtAs. The insonation of the arteries was performed in its proximal third, with a maximal angle of 60°. The calculation of the PI of the UtA was made from a wave similar to at least other 3 symmetrical waves, and the mean PI of UtAs was calculated using the simple arithmetic mean between the PI values of the left and right arteries. Protodiastolic notch in uterine Doppler waveform was also recorded at the scan. We defined as abnormal Doppler velocimetry a mean UtA PI > 95th percentile as well as the presence of bilateral notching, and they reflect the higher amount of impedance to blood flow distal to the UtA.^[5,21]^ All measurements were performed with the mothers in a semirecumbent position and when there were no fetal gross body or chest movements.

### 2.2 Outcome measures

The primary outcome was considered the development of late-onset HDP with or without proteinuria. The secondary outcome was the role of fetal aIMT in the prediction of late HDP.

### 2.3 Sample size calculation and statistical analysis

The R program (version 3.1.2, R Foundation for Statistical Computing, Vienna, Austria, http://www.R-project.org/) was used to analyze the collected data. The present study was done according to TRIPOD statement.^[22]^ The target sample size was at least of 38 subjects per group and 76 subjects in total. According to previous published data, this sample size is sufficient to detect differences in mean aIMT of 0.15 between controls and cases, with a standard deviation of 0.2, 5% significant level, and power of 90%.^[5]^ The *P* value <0.05 was defined as significant considering a 2-sided alternative hypothesis. The distribution normality of variables was assessed using Kolmogorov–Smirnoff test. Data were presented by mean (±standard deviation), median and interquartile range, or percentage and absolute values. In addition, odds ratio and 95% confidence interval (CI) or a specified reference value (e.g., area under the curve [AUC]) and 95% CI were also used. During the analysis, we used the following statistical tests: in case of continuous variables, Student *t* test or Wilcoxon test; in case of categorical variables, Fisher exact test or chi-square where appropriate. Univariate and multivariate logistic regression analyses were also performed. In the multivariate logistic regression model, the development of HDP after the 34th gestational week was considered as the dependent variable, whereas fetal abdominal aorta characteristics and the other possible predictive factors were considered as independent variables. Then, in the multivariate model, all potentially influencing factors and their interactions were accommodated in a single analysis, except when the interaction term was nonsignificant (in which case we analyzed the no-interaction model). Furthermore, we included in the initial multivariate model all the factors that had a *P* value <0.200 in univariate analysis and then performed a stepwise selection to obtain the final multivariate logistic regression model. In addition, model performance was assessed by the area under the receiver operator curve with the relative 95% CI. Bonferroni correction was applied to multivariate analysis. Using the multivariate logistic regression model, a nomogram was developed as a prognostic scoring system incorporating significant ultrasound and clinical variables including fetal abdominal aorta data available at ultrasound examination of 29 to 32 weeks’ gestation. Points for variables in the nomogram were determined by their logistic regression model coefficients. In addition, also a risk calculator based on the same logistic regression model coefficients was made. Thereafter, a nomogram calibration plot was drawn using the same regression coefficients of multivariate logistic model and 1000 boot strap repetitions to implement internal validation of our nomogram.

## 3 Results

During the study period, 1538 singleton pregnant women underwent ultrasound examination at 29 to 32 weeks’ gestation. A total of 1381 pregnancies were included in the study, while 157 were excluded because they presented with at least one of the exclusion criterion. Seventy-eight patients presented a preterm delivery. In 73 cases, a diagnosis of HDP after 34 weeks’ gestation was made, while in 1381 cases the pregnancy was regular.

In Table 1, the characteristics of the studied population are described. Women affected by late-onset HDP were older, with a higher BMI, and more frequent nulliparous. The blood pressure of cases was higher than the controls (*P* < 0.05). Moreover, patients affected by HDP delivered earlier and more frequently by cesarean section than controls (*P* < 0.05). Notwithstanding, mean neonatal weight was lower in the late-onset HDP group, the neonatal weight percentiles were similar and not significantly different (*P* = 0.97). However, in the group of women affected by late-onset HDP, there was an increased prevalence of SGA (37.0%, 27/73) in comparison with 8.7% of controls (114/1308) (*P* < 0.05). Furthermore, we also found a nonsignificant increase in the prevalence of LGA in the group of patients affected by HDP (*P* = 0.08). Table 2 shows the data relative to the ultrasound examination performed at 29 to 32 weeks’ gestation. In the HDP group, we found an increased aIMT, fetal abdominal aorta PI, UA PI, maternal UtA mean PI, maternal UtA mean RI, and prevalence of bilateral notch in UtAs. Likewise, we found also a decreased mean kidney volume (Table 2).

**Table 1:**
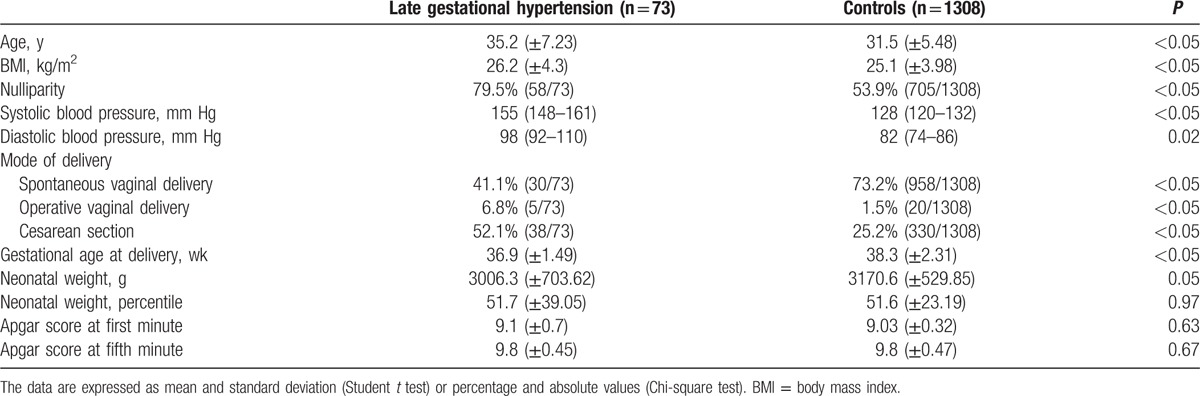
Population characteristics.

**Table 2:**
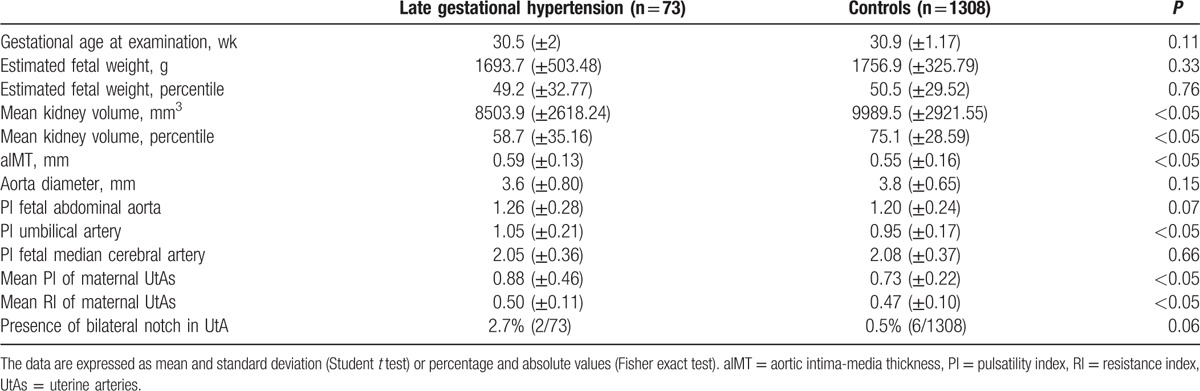
Ultrasound examination at 29 to 32 weeks’ gestation.

Table 3 explains the univariate and multivariate logistic regression analyses. After stepwise selection, the following variables were included in the final model: maternal age, prepregnancy BMI, nulliparity, aIMT, UA PI, and mean maternal UtA PI. In particular, increased maternal age, nulliparity, increased aIMT, increased UA PI, and mean PI of maternal UtAs were significant risk factors for late HDP development (*P* < 0.05). Moreover, the multivariate model intercept was −19.73.

**Table 3:**
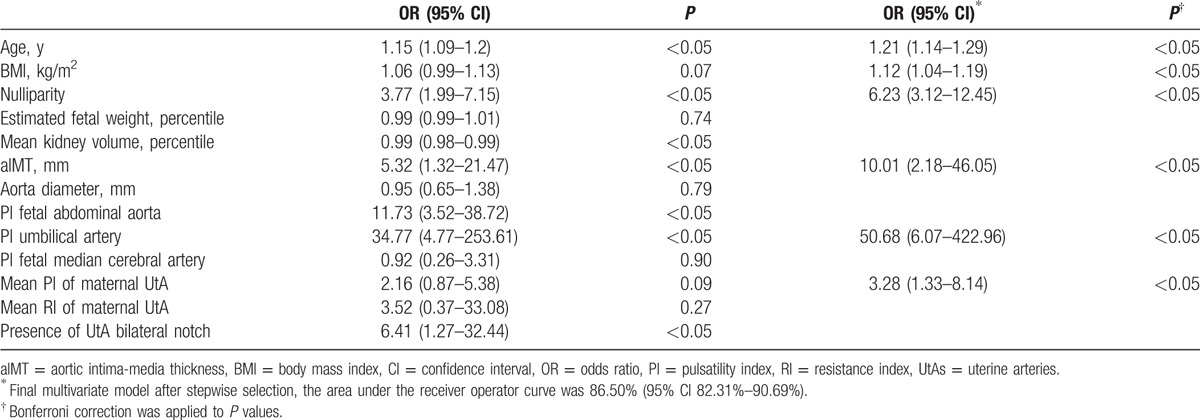
Univariate and multivariate logistic regression analysis.

**Figure 1:**
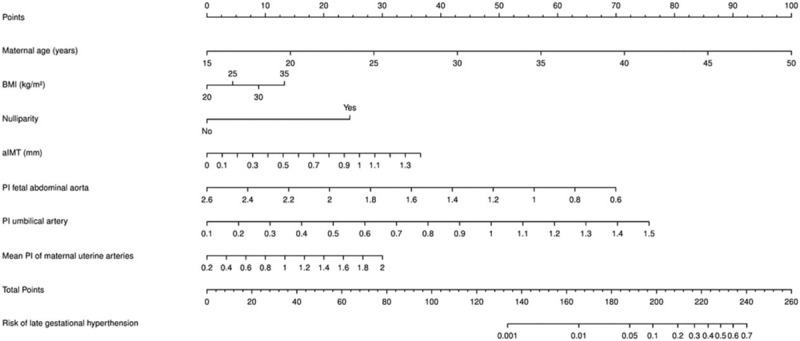
Nomogram for estimating the probability of late gestational hypertension development at ultrasound examination of 29 to 32 weeks’ gestation by fetal abdominal aorta biometry, maternal fetal Doppler, fetal biometry, and clinical data. Instructions for reading the nomogram: locate the age of the pregnant women on the woman's age axis, then draw a straight line up to the points axis to determine how many points the patient receives for age. Repeat the process for all other predictors. Thereafter, sum the points achieved for each predictor and locate this sum on the total points axis at the bottom of the plot. Finally, draw down a line straight to the probability of late gestational hypertension development axis. This plot will also highlight the variables with the greatest discriminatory value that are those with the widest point range in the nomogram (refer to Supplemental file 1 for excel sheet version).

**Figure 2:**
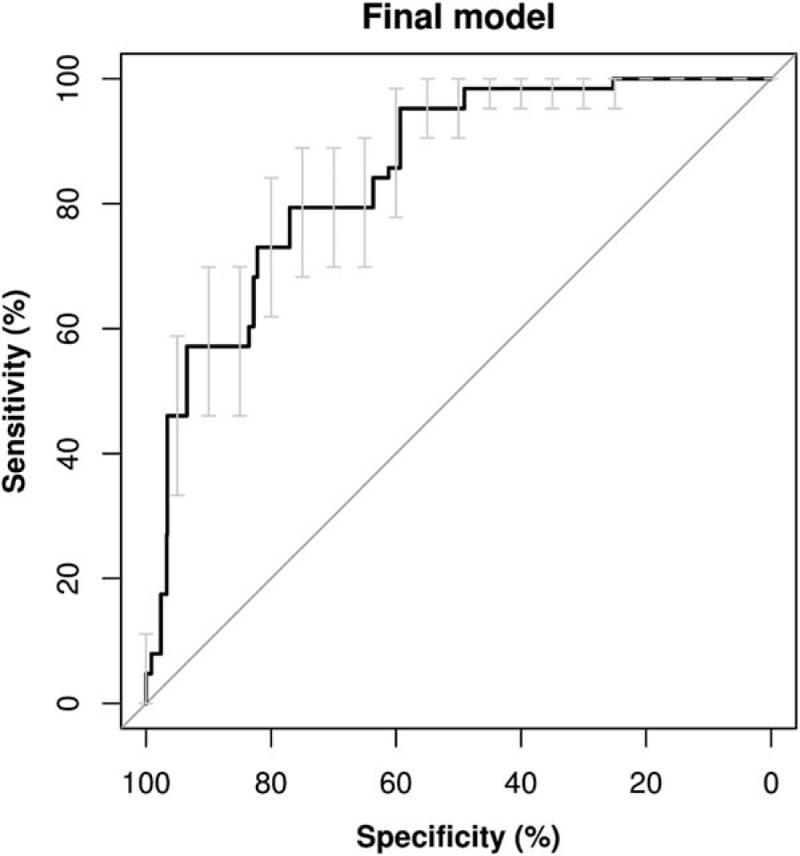
Receiver operator characteristics curve with confidence interval bars of the final multivariate logistic regression model (area under the curve was 81.07%, and the 95% confidence interval was 75.83%–86.32%).

Figure 1 represents the developed nomogram, and in the supplementary file we prepared a risk calculator. Figure 2 shows the receiver operator characteristic curve of the final multivariate logistic model that presented an AUC of 81.07% (95% CI, 75.83%–86.32%). Considering the included factors in the multivariate logistic regression model, the AUC values were for maternal age 65.85% (95% CI, 57.4%–74.29%), for prepregnancy BMI 59.99% (95% CI, 51.32%–68.66%), for nulliparity 63.99% (95% CI, 58.9%–69.08%), for aIMT 60.65% (95% CI, 54.37%–66.94%), for UA PI 63.77% (95% CI, 56.56%–70.99%), and for mean UtA PI 52.13% (95% CI, 43.00%–61.27%).

Internal validation of the prediction nomogram was performed using 1000 bootstrap resamples and showed satisfactory predictive ability for our model (Supplemental figure 1).

## 4 Discussion

Hypertensive disorders during pregnancy are a major cause of maternal and fetal morbidity and mortality. However, an effective prediction and monitoring might reduce fetal and maternal morbidities. The study found that fetal aIMT as well as fetal UA PI, maternal age, maternal prepregnancy BMI, parity, and mean PI of maternal UtAs assessed at ultrasound examination of 29 to 32 weeks’ gestation are significant and independent predictors for the development of HDP after 34 weeks’ gestation.

In previous studies, ultrasound-based measurement of aIMT in fetuses of mothers with late-onset HDP and IUGR fetuses seem to represent a sign of in utero preclinical endothelial remodeling. Therefore, it possibly might result in subsequent glomerulosclerosis, hypertension in young children, and atherosclerosis in adult life.^[5,19,23–27]^ These fetal features are in accordance with the Barker hypothesis that suggests an in utero imprinting that predisposes to the development of chronic diseases in later life.^[28]^ Furthermore, in this study, we demonstrated a usefulness of fetal aIMT assessed at the 29 to 32 weeks’ gestation ultrasound examination to forecast the development of late-onset HDP after 34 weeks’ gestation. In addition, a higher mean PI and RI with a bilateral notch of UtAs at the third trimester scan are known to be signs of placental dysfunction.^[5]^ Women at risk of adverse pregnancy outcomes are generally identified based on their clinical history.^[29]^ Screening by maternal history alone will detect a third of women who will develop HDP, but is ineffective in nulliparous women, who are at particular risk of this complication. Multiple biochemical markers have been studied individually and in combination as potential markers for adverse pregnancy outcomes.^[30]^

Algorithms combining maternal demographic characteristics as well as medical and obstetric history together with biophysical and biochemical markers at 30 to 34 weeks’ gestation have been developed for the prediction of adverse pregnancy outcomes.^[31–34]^ In our study, we aimed to assess the development of HDP arising after 34 weeks’ gestation by collating information collected at 29 to 32 weeks’ gestation ultrasound scan, including aIMT measurement that was never considered until now for such prediction algorithms.

The final multivariate model presented in this study that considers as predictors fetal aIMT, fetal UA PI, maternal age, maternal prepregnancy BMI, parity, and mean PI of maternal UtAs in the development data presented an AUC of 81.07% (95% CI, 75.83%–86.32%). In addition, in this model, the detection rate, at 10% false positive rate, was 57.1% for late HDP (Fig. 2). Despite the low specificity of this prediction model, it is easy to implement a prediction method based on clinical information and ultrasound examination assessment. A different maternal and fetal concentration of soluble fms-like tyrosine kinase-1 (sFlt-1) and vascular endothelial growth factor has been described in presence of hypertensive disorders, leading to endothelial dysfunction, hypertension, and proteinuria in animal models.^[35,36]^ According to these results, fetal aIMT could be the vascular sign of biochemical imbalance and placental insufficiency, and might be considered in more studies on late HDP prediction with ultrasound examination parameters. In previous published studies, about third-trimester screening of pregnancies complications and adverse perinatal events, sFlt-1 was considered but aIMT never before.^[32–34,37,38]^ The validated models all included UtA Doppler measures or biomarkers that require laboratory testing that is not currently part of routine practice and is too expensive for use in low- or middle-income countries.^[39]^

Therefore, in previous studies, the detection rate, at 10% false positive rate, was 75% for term pre-eclampsia,^[33,34]^ while in our study it was 57.1%. However, in contrast to other studies, the ultrasound predictors in this study were assessed earlier, thus at 29 to 32 weeks’ gestation and not at 30 to 34 weeks’ gestation,^[32–34,37,38]^ and it is known that it is easier to predict the negative outcomes closer to the time when the predictors have been assessed.^[40]^ Therefore, the low detection rate at 10% false positive rate was probably partly due to the early assessment of predictors than in previously published studies.

The main limitation of the study is the unavailability of a second population to validate the model, although an internal validation was performed. Among the strengths of this study, we have a prospective collection of data and the assessment of fetal aIMT for the first time as predictor for late-onset HDP.

## 5 Conclusion and implications

Current clinical opinion considers mothers presenting with mild-to-moderate hypertension to have obstetric outcomes not significantly different from those of the normal population. However, information regarding the long-term outcomes of pregnancies complicated by mild-to-moderate maternal hypertension is sparse. The potential clinical use of the present model is to assess the risk of late HDP development at 29 to 30 weeks’ gestation ultrasound examination, in order to select and closely monitor high-risk pregnancies. The usefulness of selecting high-risk women for late HDP development resides in the possibility to intensively monitor maternal and fetal wellbeing of a selected group of pregnancies to allow an earlier diagnosis for mitigating eventual adverse outcomes by means of medical induction of labor.^[12]^ However, this preliminary model needs to be validated in other populations and improved by future studies in the second trimester of pregnancy and also during childhood. The fetal parameter aIMT has for the first time a role in a predictive model for gestational hypertension. In addition, fetal aIMT in future studies should be considered for implementing routine ultrasound examinations as a possible predictor for maternal and fetal outcomes, mainly because it can be considered an early sign of fetal endothelial remodeling.^[5,19]^

## Supplementary Material

Supplemental Digital Content

## Supplementary Material

Supplemental Digital Content
