# Supplementary material for: A possible new approach in the prediction of late gestational hypertension: The role of the fetal aortic intima-media thickness
Source: Medicine (Baltimore). 2017 Jan 13;96(2):e5515. doi: 10.1097/MD.0000000000005515 (PMC5266153; doi:10.1097/MD.0000000000005515)

**Supplemental Figure 1**

Calibration plot of the developed nomogram that shows on x-axis the predicted probability and on y-axis is the actual probability of late gestational hypertension development. Moreover, dotted line represents the location of the ideal nomogram, in which the predicted and actual probabilities are identical; broken line represents the actual nomogram performance without correction for overfitting; and solid line shows the bootstrap-corrected performance of the developed nomogram.

In addition, mean absolute error was equal to 0.015, mean squared error was equal to 0.00052 and 0.9 Quantile of absolute error was equal to 0.044.


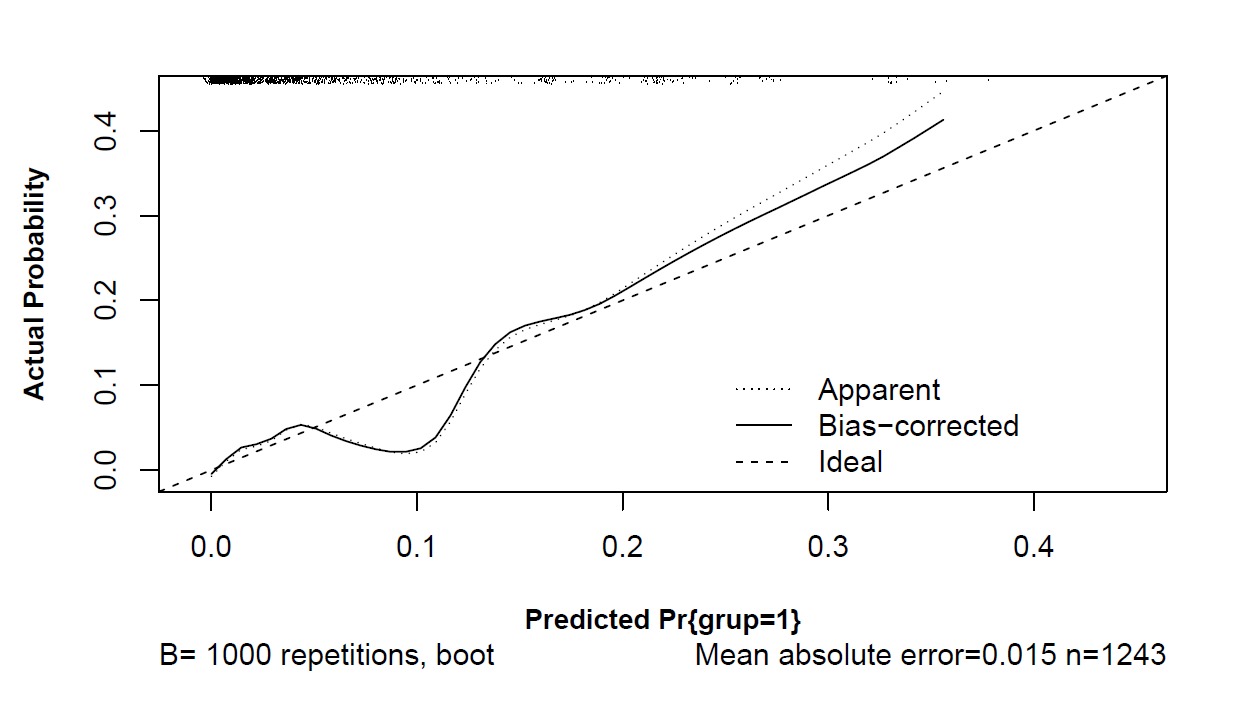

Supplement: Supplemental Digital Content [file medi-96-e5515-s002.doc]
